# Multiparametric Evaluation of Drug-Induced Acute Kidney Injury Using Preclinical 7T Magnetic Resonance Imaging in Rat Models

**DOI:** 10.3390/metabo15090593

**Published:** 2025-09-07

**Authors:** Tomohiro Natsuyama, Junpei Ueda, Isamu Yabata, Reika Sawaya, Koji Itagaki, Shigeyoshi Saito

**Affiliations:** 1 Department of Medical Physics and Engineering, Division of Health Sciences, Graduate School of Medicine, The University of Osaka, Suita 560-0871, Osaka, Japanuedaj@sahs.med.osaka-u.ac.jp (J.U.); u010443b@ecs.osaka-u.ac.jp (R.S.); beitou@kuhp.kyoto-u.ac.jp (K.I.); 2 Department of Radiological Sciences, Faculty of Health Sciences, Morinomiya University of Medical Sciences, Osaka 559-8611, Osaka, Japan; 3 Department of Radiology, The University of Osaka Hospital, Suita 560-0871, Osaka, Japan; 4 Division of Clinical Radiology Service, Kyoto University Hospital, Kyoto 606-8507, Kyoto, Japan; 5 Department of Advanced Medical Technologies, National Cerebral and Cardiovascular Center, Suita 564-8565, Osaka, Japan; 6 Immunology Frontier Research Center, The University of Osaka, 3-1 Yamadaoka, Suita 560-0871, Osaka, Japan; 7 World Premier International Research Center Initiative Premium Research Institute for Human Metaverse Medicine, The University of Osaka, 2-2 Yamadaoka, Suita 565-0871, Osaka, Japan

**Keywords:** 7T magnetic resonance imaging, acute kidney injury, gentamicin, T_1_ map, T_2_ map, kidney

## Abstract

**Objectives**: Acute kidney injury (AKI), characterized by a rapid decline in renal function, affects approximately 13 million new patients annually. Adverse drug reactions have increasingly contributed to renal injury, underscoring the need for methods to directly and quantitatively evaluate renal injury. **Methods**: We utilized a drug-induced AKI model using gentamicin overdose, combining 7T magnetic resonance imaging (MRI) relaxation time measurements and blood tests to evaluate pathophysiological changes from multiple perspectives. Ten-week-old Wistar rats received intraperitoneal administration of gentamicin (80 mg/kg) for 7 days. Under respiratory synchronization, T_1_, T_1rho_, T_2_, and T_2_* maps were obtained in six control and five disease model rats. Relaxation times in the cortex and medulla were measured separately and compared between groups. **Results**: Blood tests evaluated Na, K, Cl, blood urea nitrogen, creatinine, and hematocrit levels. Renal tissue damage was evaluated via hematoxylin and eosin (HE) staining. Relaxation time showed significant changes in the cortex, especially in the T_1_ (control: 1156.7 ± 140.0, gentamicin: 1550.4 ± 162.1, *p* < 0.05) and T_2_ (control: 42.9 ± 3.4, gentamicin: 53.4 ± 4.8, *p* < 0.05) maps. Blood tests revealed significant increases in Na, blood urea nitrogen, creatinine, and hematocrit levels in the disease model. A correlation was observed between the T1 map of the renal cortex and each substance. HE staining revealed tissue damage due to renal injury. **Conclusions**: Multiparametric MRI facilitates quantitative and multidimensional evaluation of renal pathological changes caused by drug-induced AKI.

## 1. Introduction

Acute kidney injury (AKI) is a rapid decline in renal function, evidenced by a decrease in the glomerular filtration rate and increased blood levels of nitrogen compounds, such as creatinine and urea nitrogen [1,2,3,4,5,6]. AKI affects more than 13 million individuals worldwide annually [7], with 5–20% of hospitalized patients developing the condition—a prevalence that increases yearly [2,8,9,10,11,12,13]. Risk factors for AKI include drug side effects, aging, ischemia, and other serious illnesses [1,5,8,11,13,14,15,16,17,18]. Among these, drug-induced AKI, where renal function declines owing to drug side effects, has become a growing concern. Drug-induced AKI occurs in 14–26% of adults in a prospective cohort study [19], especially in China, where drug-induced AKI is becoming a major etiology of AKI [18]. AKI is more likely to occur because of the high blood flow and metabolic activity of the kidneys [6], and renal excretion is the main method of elimination [15,18,20]. Drug-induced AKI primarily affects glomerular filtration and tubular secretion in the kidneys, resulting in tubular damage [20].

Gentamicin induces drug-induced AKI. Gentamicin is an aminoglycoside antibiotic used to treat Gram-negative bacterial infections [5,11,12,18,21,22,23]. Similar to common drug-induced AKI, gentamicin-induced AKI persists in the proximal tubules and causes oxidative stress and tubular damage [5,11,18,22]. This injury is mainly caused by the cortical and outer stripes of the outer medulla (OSOM) of the kidney [6,12,21]. Gentamicin has been used as a model for nephrotoxicity in preclinical studies [6]. The common clinical methods for evaluating AKI involve measuring blood urea nitrogen (BUN) and serum creatinine; however, these methods are insensitive or nonspecific to AKI, making it impossible to properly evaluate AKI stage [3,13,17,24,25,26]. Ultrasound and computed tomography are used for the imaging diagnosis of AKI. However, ultrasound faces challenges in understanding renal structures and quantitative evaluation, and computed tomography poses the risk of radiation exposure and the use of contrast agents. Therefore, magnetic resonance imaging (MRI), a noninvasive and quantitative imaging method for evaluating AKI, is considered useful in clinical practice.

Multiparametric MRI is a multidimensional quantitative method used to evaluate structural and functional aspects of the kidney. Multiple MRI parameters can be obtained in a single scanning session, making it possible to evaluate the overall kidney structure, vascular dynamics, kidney function, and microstructure. Multiparametric MRI is a promising “biomarker imaging” technique because it can noninvasively assess the progression and stage of AKI with high accuracy without contrast [4,7,9,24,27]. Currently, T_1_ and T_2_ maps are relaxation time measures used for renal MRI in preclinical studies. The T_1_ map of the kidney is increased by inflammation, edema, and leakage of tissue fluid due to cell swelling and fibrosis [7,13,17,24,28,29,30,31,32]. This change is more common in the cortex and OSOM because the tubules are clustered in these areas [7,13,24]. Wu et al. showed that increased T_1_ values in the renal cortex positively correlated with glomerular, tubular, and vascular injury scores, indicating that the T_1_ map is useful for assessing renal pathology [28]. Additionally, Hueper et al. argued that the T_1_ map can confirm kidney volume loss due to renal injury and predict renal outcomes when renal histology cannot distinguish AKI severity [17]. The T_2_ map is sensitive to changes in tissue water content and is useful for assessing capillary leakage and tissue edema in the kidney [24,25,30,33,34,35]. Wang et al. suggested that the T_2_ map may be more sensitive in identifying adverse effects due to AKI by targeting kidney water content [13]. Similar to the T_1_ map, the T_2_ map also shows the increased signal in the renal cortex and OSOM because of AKI [24,25,33,35]. Thus, the number of renal MRI studies has recently increased. Historically, renal MRI studies of AKI have often relied on single-parameter readouts—for example, native T_1_ mapping alone in ischemia-induced AKI [17] or BOLD/T_2_* alone in gentamicin nephrotoxicity [21]—or on limited pairwise combinations such as T_2_ mapping with ADC [33]. By contrast, comprehensive multiparametric protocols have emerged only recently and have been applied primarily to ischemia–reperfusion or transplantation models [4,9,24,34,35], underscoring the novelty of our non-contrast, multiparametric approach in drug-induced AKI.

Beyond the widely used native T_1_ and T_2_ maps, we included T_1rho_ and T_2_* because they interrogate complementary biophysical compartments that are directly relevant to drug-induced AKI. T_1rho_ mapping measures spin-lock relaxation arising from low-frequency interactions between water and macromolecules, and increases with macromolecular content, proteoglycan/collagen deposition, and extracellular matrix remodeling—processes linked to inflammation and evolving fibrosis [36,37,38]. In contrast, T_2_* is sensitive to microscopic magnetic-susceptibility differences, particularly those created by deoxyhemoglobin, thereby reporting on microvascular oxygenation (BOLD) and hemodynamic status, while also reflecting changes in tissue water content and tubular volume [31]. Because gentamicin predominantly injures proximal tubules and the cortex/outer medulla, leading to edema, tubular dilatation, and matrix alterations [6,12,18], we hypothesized that T_1rho_ and T_2_* would complement T_1_/T_2_ by capturing macromolecular and oxygenation-related aspects of injury without exogenous contrast. This rationale motivated their inclusion in our multiparametric protocol.

In this study, drug-induced AKI rat models were used to perform four relaxation time measurements: T_1_ and T_2_ maps, which are commonly used for AKI evaluation, and T_1rho_ and T_2_* maps. This study aimed to evaluate the pathophysiology of drug-induced AKI in a multiparametric manner by combining relaxation time measurements with blood tests.

## 2. Materials and Methods

### 2.1. Animal Preparation

The Research Ethics Committee of our University approved all experimental protocols. All experimental procedures involving animals and their care were performed in accordance with the Osaka University Guidelines for Animal Experimentation and the National Institutes of Health Guide for the Care and Use of Laboratory Animals. This animal study was conducted in accordance with the ARRIVE guidelines. Animal experiments were performed using 9–10-week-old (209–255 g) male Wistar rats purchased from Japan SLC (Hamamatsu, Japan). All rats were housed in a controlled vivarium environment (24 °C; 12/12 h light/dark cycle) and were fed a standard pellet diet and water ad libitum. We intraperitoneally administered gentamicin at 80 mg/kg/day to six Wistar rats for 7 days [21]. This dosing regimen (80 mg/kg/day for 7 days) has been widely used in preclinical rat models and is reported to induce consistent renal tubular injury and impaired renal function without excessive mortality [6]. Here, the gentamicin-induced AKI model is referred to as the “gentamicin model.” MRI experiments were performed on six control and six gentamicin model rats (one of which died during the experiment) at 10 weeks of age. Data were obtained from six control and five gentamicin-treated rats.

### 2.2. MRI Equipment

MRI scans of animal kidneys were acquired using a horizontal 7T scanner (PharmaScan 70/16 US; Bruker Biospin, Ettlingen, Germany) equipped with a volume coil with an inner diameter of 60 mm. To obtain an MRI, the rats were positioned in a stereotaxic frame to prevent movements during acquisition [39,40]. The body temperature of the rats was maintained at 36.5 °C with regulated water flow and continuously monitored using a physiological monitoring system (SA Instruments Inc., Stony Brook, NY, USA). All kidney MRI procedures on rats were performed under general anesthesia induced with isoflurane (Viatris Inc., Tokyo, Japan, 3.0% for induction and 2.0% for maintenance). All rats were euthanized using 5% isoflurane after MRI scanning. In order to quantify native relaxation parameters without altering tissue relaxation properties or imposing additional renal burden in the AKI setting, no exogenous contrast agents were administered during any MRI acquisition.

Fitting for mapping: Under respiratory gating, T_2_-weighted images were acquired with the rapid acquisition with relaxation enhancement (RARE) with the following parameters: TR = 730; TE = 24 ms; RARE factor = 8; slice thickness = 2 mm; field-of-view = 60 × 60 mm^2^; matrix size = 234 × 234; slice number =1; slice orientation = trans-coronal; resolution = 234 μm × 234 μm; and scan time = 1 min, 33 s.

Under respiratory gating, T_1_ mapping images were acquired with the RARE with variable TR with the following parameters: TR = 200, 400, 800, 1500, 3000, 5500; TE = 16 ms; RARE factor = 4; slice thickness = 2 mm; field-of-view = 60 × 60 mm^2^; matrix size = 128 × 128; slice number =1; slice orientation = trans-coronal; resolution = 469 μm × 469 μm; and scan time = 6 min, 48 s.

Under respiratory gating, T_1rho_ mapping images were acquired with the spin-lock RARE with the following parameters: TR = 2500; TE = 30 ms; RARE factor = 8; spin-lock frequency = 460 Hz; time of spin-lock = 7.8, 17.8, 27.8, 37.8, 47.8, and 57.8 ms; slice thickness = 2 mm; field-of-view = 60 × 60 mm^2^; matrix size = 160 × 160; slice number = 1; slice orientation = trans-coronal; resolution = 375 μm × 375 μm; and scan time = 5 min. The TR (2500 ms), TE (30 ms), and spin-lock frequency (460 Hz) parameters were chosen based on previously reported 7T small animal protocols [36].

Under respiratory gating, T_2_ mapping images were acquired with the multi-slice multi-echo sequence with the following parameters: TR = 2200; TE = 7.5, 15, 22.5, 30, 37.5, 45, 52.5, 60, 67.5, 75, 82.5, and 90 ms; RARE factor = 12; slice thickness = 2 mm; field-of-view = 60 × 60 mm^2^; matrix size = 160 × 160; slice number =1; slice orientation = trans-coronal; resolution = 375 μm × 375 μm; and scan time = 5 min, 52 s.

Under respiratory gating, T_2_* mapping images were acquired with the multi-gradient echo sequence with the following parameters: TR = 800; TE = 4.5, 10, 15.5, 21.5, 27, 32.5, 38, and 43.5 ms; RARE factor = 8; slice thickness = 2 mm; field-of-view = 60 × 60 mm^2^; matrix size = 160 × 160; slice number =1; slice orientation = trans-coronal; resolution = 375 μm × 375 μm; and scan time = 3 min, 12 s.

### 2.3. MRI Data Analysis

Each mapping image (T_1_, T_1rho_, T_2_, and T_2_* maps) of six control model rats and five gentamicin-treated model rats was analyzed. T_1_, T_1rho_, T_2_, and T_2_* values were measured separately in the renal cortex and medulla by enclosing the region of interest and measuring each value. In this study, the renal medulla refers to the ISOM. This approach was employed because of the difficulty in measuring the OSOM uniformly using all the relaxation time measurement methods. Additionally, T_2_-weighted images were used to compare kidney volumes. Kidney volume was defined as the calculated area of the kidney in the T_2_-weighted image surrounded by the region of interest multiplied by the slice thickness (2 mm).

### 2.4. Blood Test

After the MRI experiment, blood was collected from the tail veins of the rats and tested using a blood test kit (i-STAT; Abbott Japan LLC, Tokyo, Japan). The target substances were Na, K, Cl, BUN, creatinine, and Hct values.

### 2.5. Histological Analysis

After the MRI experiment, the control and gentamicin model rats were sacrificed, and both kidneys were removed for HE staining. We asked the Sapporo General Pathology Laboratory to perform HE staining of the kidneys. We observed the histomorphology of the kidneys in each HE-stained rat model.

### 2.6. Statistical Analysis

Data are presented as mean ± standard deviation. Parametric t-tests were used to compare kidney T_1_, T_1rho_, T_2_, and T_2_* values. All analyses were performed using the Prism 8 software (GraphPad Software, Solana Beach, CA, USA). Statistical significance was set at *p* < 0.05. Correlations between relaxation times and blood test parameters were evaluated using Pearson’s correlation coefficient (r). Strength of correlation was described using standard conventions: r = 0.1–0.3 (weak), r = 0.3–0.5 (moderate), and r ≥ 0.5 (strong).

## 3. Results

### 3.1. Comparison of Body Weight and Kidney Volume

**Figure 1:**
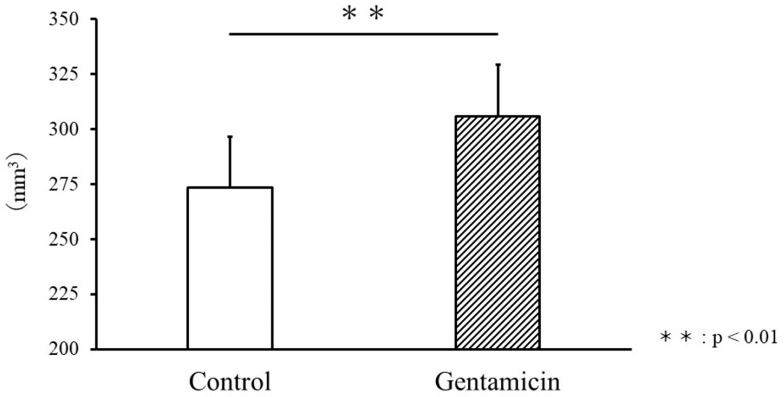
Comparison of kidney volume between the control and gentamicin groups. A significant increase was observed in kidney volume in the gentamicin model group (*p* < 0.01).

The average weight of rats in the control group was 247.3 ± 7.6 g. In contrast, the average weight of rats in the gentamicin group was 220.2 ± 11.7 g. Kidney volume was 273.5 ± 23.2 mm^3^ in the control group compared with 305.8 ± 23.5 mm^3^ in the gentamicin group (*p* < 0.01, Figure 1), confirming renal enlargement due to AKI. Figure 1 shows the comparison of kidney volumes, which were significantly greater in the gentamicin group (*p* < 0.01). Of the six gentamicin-treated rats, five completed the study. One rat died during the final scanning session, and we attribute this event to a combination of gentamicin toxicity and anesthetic risk, consistent with previous reports of mortality in aminoglycoside nephrotoxicity studies.

### 3.2. Blood Test

**Figure 2:**
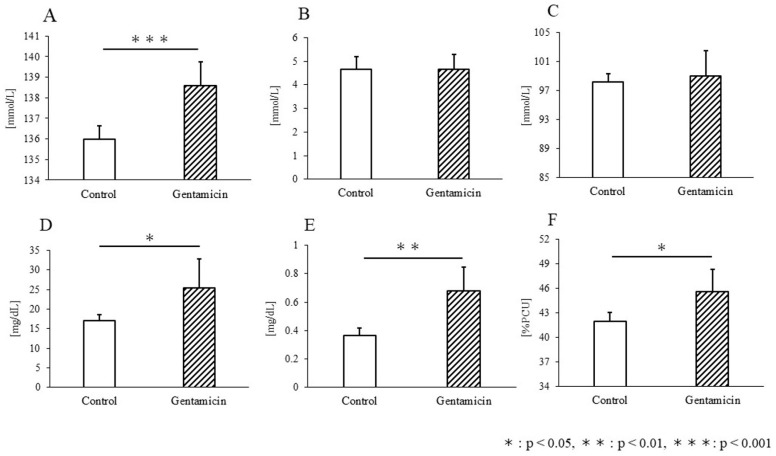
(**A**–**F**) Show the comparison of Na (**A**), K (**B**), Cl (**C**), BUN (**D**), Crea (**E**), and Hct (**F**) between the control and gentamicin groups. Na was significantly increased in the gentamicin group (*p* < 0.001), but no significant change was observed for K or Cl. Significant increases were observed in BUN (*p* < 0.05), creatinine (*p* < 0.01), and hematocrit (*p* < 0.05) in the renal disease model group (**D**–**F**).

Figure 2 compares Na, K, Cl, BUN, creatinine, and hematocrit (Hct) levels between the control and gentamicin groups. For Na levels, 136.0 ± 0.6 mmol in the control group and 138.6 ± 1.1 mmol in the gentamicin group confirmed a significant increase in the disease model (*p* < 0.001, Figure 2A). This change was the largest among all the materials used in this study. K levels were 4.7 ± 0.5 mmol and 4.7 ± 0.6 mmol in the control and gentamicin groups, respectively (Figure 2B). Cl levels were 98.2 ± 1.2 mmol and 99.0 ± 3.5 mmol for the control and gentamicin groups, respectively (Figure 2C). Neither of these levels differed significantly between the disease model groups. Urea nitrogen (BUN) levels were 17.0 ± 1.7 mg/dL and 25.4 ± 7.5 mg/dL in the control and gentamicin groups, respectively, indicating a significant increase (*p* < 0.05, Figure 2D). Similarly, creatinine levels also showed a significant increase (control: 3.7 × 10^−1^ ± 0.1 mg/dL, gentamicin: 6.8 × 10^−1^ ± 0.2 mg/dL, *p* < 0.01, Figure 2E). Hct levels also increased significantly in the disease model: 42.0 ± 1.1%PCU and 45.6 ± 2.7%PCU in the control and gentamicin groups, respectively (*p* < 0.05, Figure 2F).

### 3.3. T_1_ Relaxation Time

**Figure 3:**
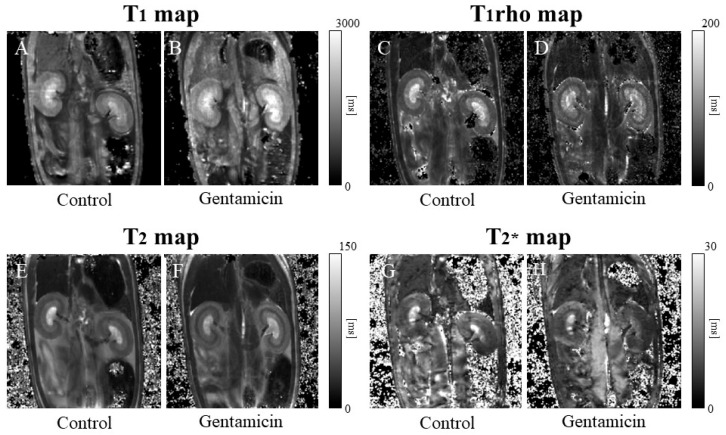
(**A**–**H**) Magnetic resonance imaging of the T_1_, T_1rho_, T_2_, and T_2_* maps. (**A**,**B**) The T_1_ map shows a kidney T_1_ value increase in the gentamicin model (**B**). (**C**,**D**) The T_1rho_ map did not show significant changes in T_1rho_ values. (**E**,**F**) The T_2_ map shows increased T_2_ values due to kidney injury, and the outer stripe of the outer medulla was visible (**F**). (**G**,**H**) The T_2_* map did not change significantly in the gentamicin model (**H**). The scale bar on the T_1_ map shows 0 ms to 3000 ms. The scale bar on the T_1rho_ map shows 0 ms to 200 ms. The scale bar on the T_2_ map shows 0 ms to 150 ms. The scale bar on the T2 star map shows 0 ms to 30 ms.

**Figure 4:**
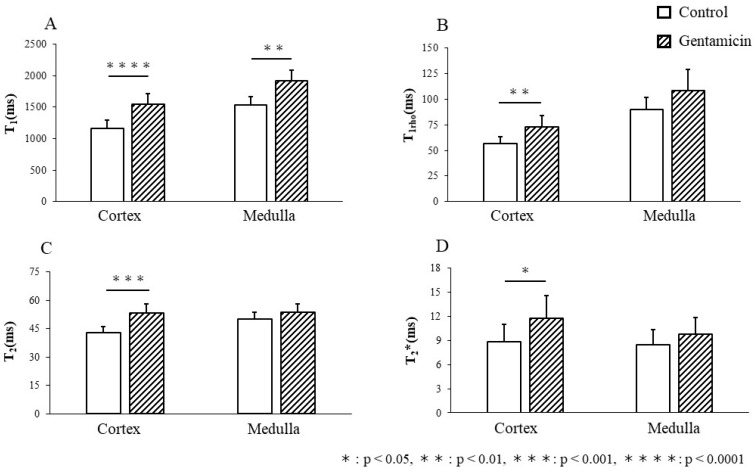
T_1_ values (**A**) were increased in the renal cortex (*p* < 0.0001) and medulla (*p* < 0.001). T_1rho_ value (**B**), T_2_ value (**C**), and T_2_* value (**D**) showed no significant changes in the renal medulla, but all showed significant increases in the renal cortex (T_1rho_ map: *p* < 0.01, T_2_ map: *p* < 0.001, and T_2_* map: *p* < 0.05). Significant increases were observed in T_1_ and T_2_ values in the renal cortex (**A**,**C**).

Figure 3A–H show typical MRI relaxation maps, and Figure 4A–D show the values of all MRI relaxation maps. The T_1_ values of the kidneys in the control group were 1156.7 ± 140.0 ms and 1531.1 ± 139.5 ms for the renal cortex and medulla, respectively (Figure 3A). In contrast, the T_1_ values of the kidneys in the gentamicin group were 1550.4 ± 162.1 ms and 1918.5 ± 168.8 ms for the renal cortex and medulla, respectively (Figure 3B). Renal injury significantly increased the T_1_ value in the renal cortex (*p* < 0.0001) and medulla (*p* < 0.01), with a greater increase in the renal cortex (Figure 4A).

### 3.4. T_1rho_ Relaxation Time

The T_1rho_ values of the kidneys in the control group were 56.7 ± 6.9 ms and 90.2 ± 11.7 ms for the renal cortex and medulla, respectively (Figure 3C). In contrast, the T_1rho_ values of the kidneys in the gentamicin model group were 73.0 ± 11.3 ms and 108.7 ± 20.4 ms for the renal cortex and medulla, respectively (Figure 3D). In the AKI model, a significant increase was observed in renal T_1rho_ values in the renal cortex (*p* < 0.01), but not in the renal medulla (Figure 4B).

### 3.5. T_2_ Relaxation Time

The T_2_ values of the kidneys in the control model group were 42.9 ± 3.4 ms and 50.1 ± 3.8 ms for the cortex and renal medulla, respectively (Figure 3E). In contrast, the T_2_ values of the kidneys in the gentamicin group were 53.4 ± 4.8 ms and 53.5 ± 4.4 ms for the cortex and renal medulla, respectively (Figure 3F). Similar to the T_1rho_ value, the T_2_ value showed a significant increase only in the renal cortex (*p* < 0.001); however, the difference was greater than that of the T_1rho_ value (Figure 4C).

### 3.6. T_2_* Relaxation Time

The T_2_* values of the kidneys in the control group were 8.9 ± 2.1 ms and 8.5 ± 1.8 ms for the renal cortex and medulla, respectively (Figure 3G). In contrast, the T_2_* values of the kidneys of the gentamicin group were 11.7 ± 2.8 ms and 9.7 ± 2.1 ms for the renal cortex and medulla, respectively (Figure 3H). Thus, a significant increase was observed in T_2_* values in the renal cortex of the gentamicin group compared with the control group (*p* < 0.05). No significant differences were observed in the renal medullae (Figure 4D).

### 3.7. Histological Staining

**Figure 5:**
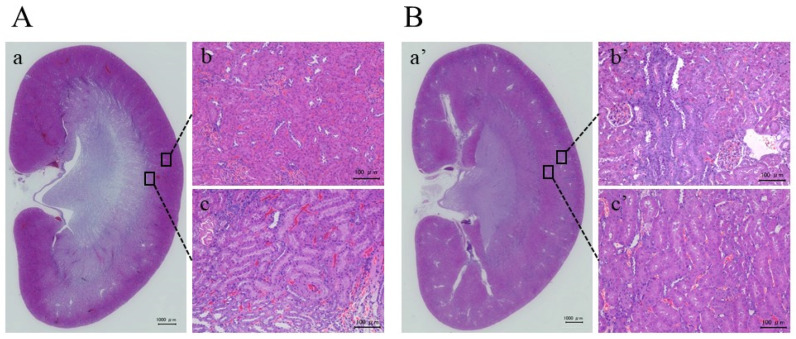
Hematoxylin and eosin stain of rat kidney from the control (**A**) and gentamicin (**B**) models. A-a shows the entire kidney of the control model, A-b is a close-up image of the cortex, and A-c is a close-up image of the medulla. Similarly, B-a’ shows the entire kidney of the gentamicin model, B-b’ is a close-up of the cortex, and B-c’ is a close-up of the medulla. In disease models, the entire cortex was injured (B-a’). A close-up view of the cortex shows that the intercellular spaces have expanded owing to vacuolation and thinning of the plasma membrane (B-b’).

All rats were euthanized by 5% isoflurane after an MRI scan. We removed the kidneys from the control and disease models and performed hematoxylin and eosin (HE) staining in Figure 5A,B. Normal kidneys in the control model had closely packed cells (Figure 5A-a); however, those in the disease model had an overall injury that was particularly obvious in the cortical area (Figure 5B-a’). Furthermore, when the cortex and medulla were expanded, the glomeruli were atrophied and vacuolated in the disease model compared with the control model. Additionally, the cell membranes were thinner, indicating that the intercellular spaces were enlarged. Tubular dilatation was also observed with occasional cell debris inside the cortical tubules (Figure 5A-b,A-c,B-b’,B-c’).

### 3.8. Correlation of Relaxation Time and Blood Tests

Table 1 shows the correlation between the values of the substances targeted in the blood tests and relaxation times in the cortex (Table 1A) and medulla (Table 1B). Particularly, the T_1_ map of the renal cortex was strongly and positively correlated with Na (r = 0.75), BUN (r = 0.68), Crea (r = 0.70), and Hct (r = 0.83) (Table 1A). According to standard conventions (r ≥ 0.5 = strong), these associations were classified as strong. The levels of these substances were significantly increased in the disease model. The T_1rho_ and T_2_ maps in the cortex also correlated with Na (T_1rho_: r = 0.50, T_2_ map: r = 0.46) and BUN (T_1rho_: r = 0.57, T_2_ map: r = 0.59) (Table 1A).

## 4. Discussion

In this study, we quantitatively evaluated renal injury in a drug-induced AKI model using four different relaxation time measures (T_1_ map, T_1rho_, T_2_, and T_2_* maps) and correlated them with blood test results. To the best of our knowledge, this study is the first multiparametric evaluation of a drug-induced AKI model using these relaxation time measures in combination with blood tests. Among them, the T_1_ map showed an increase in relaxation time due to AKI. As the other relaxation time measures also showed results representing different features of renal injury, this study demonstrated that combining them in a single scanning session could provide a complementary and multifaceted assessment of renal conditions.

The T_1_ map has been used for MRI evaluation of AKI, and various reports have described its features. For example, ischemia–reperfusion-induced AKI models show a marked increase in T_1_ values in the cortical and extramedullary layers in disease models [13,17,24]. In this study, the T_1_ map of the disease model showed a significant increase in values in the cortex and medulla. As a factor in the increase in T_1_ values in AKI, the T_1_ map is sensitive to pathological changes occurring in the tissue and can detect changes in the molecular environment, such as increased renal tissue water content and deposition of macromolecular components induced by AKI [2,7,13,17,24,36,41,42,43]. Therefore, T_1_ maps can quantitatively capture inflammation, edema, increased extracellular fluid, and fibrosis [7,9,13,24,27,29,30,36]. Increased T_1_ values in the kidney cortex are positively correlated with glomerular, tubular, and vascular damage scores [28], and the increased T_1_ values in this study may be indicative of these injuries. Some studies have reported the usefulness of T_1_ maps in assessing AKI; however, these studies are few, and reports on drug-induced AKI are lacking. This study is one of the few to use T_1_ maps to quantitatively evaluate the kidney in a drug-induced AKI model. The T_1_ map correlates well with AKI severity at early time points and provides prognostic signs of renal function [17,27,41], making it a useful method of clinical observation for treatment.

Blood tests revealed increased Na, BUN, creatinine, and Hct levels. Fluctuations in blood Na levels, whether high or low, indicate renal impairment [26,44,45]. AKI often coexists with dysnatremia [45]. Na variability is a potential risk factor for AKI that may replace serum creatinine, which is less sensitive to AKI, because it is not dependent on absolute values and is identified by rapid changes in blood Na [26]. BUN and creatinine are indicators commonly used in clinical settings and preclinical renal function tests [22]. Increased BUN and creatinine in the blood indicate impaired renal function owing to decreased renal excretory capacity [9,21,22,23,25,46,47]. Its low sensitivity is a problem; however, it is widely used as a marker of impaired renal function [9,25]. Hct levels are generally reported to decrease with AKI; however, in this study, a significant increase was observed. Several mechanisms may explain this discrepancy. First, renal hypoxia may stimulate compensatory erythropoietin release. Second, fluid imbalance or dehydration could elevate Hct. Third, gentamicin-related tubular dysfunction may alter sodium and water handling. These factors suggest that Hct changes in AKI are context-dependent and should be interpreted alongside imaging biomarkers.

The results of this study showed that Na, BUN, creatinine, and Hct levels, which were significantly increased by AKI in blood tests, were positively correlated with the T_1_ map. As mentioned above, these blood test targets increase due to impaired glomerular filtration and tubular function, reflecting the effects of renal impairment. Therefore, the positive correlation between these indices and T_1_ maps strengthens the argument that T_1_ maps can be used to quantitatively evaluate drug-induced AKI.

The T_1rho_ map is a relaxation time measurement that reflects the relaxation caused by the interaction between water molecules and surrounding macromolecules [36,37] and is sensitive to the macromolecular components of the tissue [37,38]. In this study, we observed a significant increase in T_1rho_ values due to drug-induced AKI in the renal cortex. According to Rauscher et al., T_1rho_ values increase with the severity of liver fibrosis [48,49]. One reason for this increase is the deposition of collagen and other components of the extracellular matrix, and increased T_1rho_ values might reflect changes in the macromolecular composition of the extracellular matrix [36,38,49,50]. As extracellular matrix deposition is a symptom that occurs in renal fibrosis [36,50,51], T_1rho_ may show increased values in renal fibrosis. Renal fibrosis causes an increase in T_1rho_ values in the cortex and outer medulla, which is consistent with the results of this study [50]. As this study focused on an AKI model, it cannot be concluded that the T_1rho_ map reflects established fibrosis in the acute phase. Instead, it may primarily reflect early macromolecular changes in the extracellular matrix. However, the results of this study suggest that changes in renal macromolecular compounds due to AKI can be detected using the T_1rho_ map, making the T_1rho_ map a potential biomarker for drug-induced AKI. In this context, the observed T1rho prolongation is best interpreted as reflecting early extracellular matrix remodeling, a potential precursor to fibrosis rather than fibrosis itself.

One of the adverse effects of AKI on the kidneys is edema. Edema is caused by inflammation or other factors that result in abnormal water content around the interstitium and glomeruli [1,52,53]. The T_2_ map of the kidney was evaluated as a marker of renal tissue edema [25,34,35]. Increased T_2_ map values positively correlated with tissue water content, which, in turn, correlated with AKI severity [35]. T_2_ map results showed that T_2_ values in the cortex were significantly increased by renal injury, and imaging also showed significant changes in the cortical areas. The changes in the cortex were consistent with those reported in previous studies and may reflect edema. Histological findings of enlarged intercellular spaces and tubular dilatation corroborated this interpretation, linking T2 changes directly to tissue-level edema.

Additionally, HE staining for drug-induced AKI results in renal injury, including tubular dilation, cellular remnants in the tubular lumen, inflammatory cellular infiltration, and glomerular vacuolation [12,16,18,22,25]. In this study, the disease model showed glomerular atrophy and enlargement of intercellular spaces, confirming cortical-centered changes. This result is consistent with those of previous studies. Among the factors that contribute to enlarged intercellular spaces is the high water content between renal cells, possibly because of the effects of edema in the tubular interstitium and glomeruli. The increased edema-affected results in the cortex in our HE staining results were consistent with changes in T_2_ map imaging and increased relaxation time. Therefore, T_2_ maps can be used to quantitatively evaluate edema caused by drug-induced AKI.

The T_2_* map is one of the sequences used as an indicator of blood oxygen level-dependent status and is used in the preclinical field as a test of kidney function [2,31,54,55,56]. Typically, T_2_* values decrease under hypoxia due to increased deoxyhemoglobin. However, in our study, cortical T_2_ increased. This result is most plausibly explained by edema-related susceptibility homogenization and altered water distribution, which outweighed oxygenation effects in the acute setting. Thus, cortical T_2_ increases likely reflect edema and hemodynamic alterations rather than oxygenation alone.

Interpretation of T_2_* in AKI should consider model- and phase-dependence. In warm ischemia–reperfusion, cortical and medullary R_2_* rise during ischemia (i.e., T_2_* decreases), with persistent medullary elevation and cortical reversal/normalization in early reperfusion—patterns that are consistent with evolving regional oxygenation [57]. High-temporal-resolution parametric MRI further demonstrates that T_2_^*^ and T_2_ change within minutes of ischemia and early reperfusion in rats [58]. In cold I/R (transplant-relevant) AKI, T_2_* values are lower than sham across early time points and correlate with histological injury [59]. These findings, together with consensus reviews emphasizing that BOLD/T2* reflects deoxyhemoglobin-driven susceptibility yet is modulated by hydration, edema, blood volume, and flow, support a cautious reading of our cortical T_2_* increase in gentamicin-induced AKI as arising from a combination of oxygenation and edema-related susceptibility homogenization rather than oxygenation alone [54].

Buchanan et al. [9] and Francis et al. [7] reported that the onset of AKI tends to increase the overall kidney volume. In this study, kidney volumes were also compared between the control and disease models, with results consistent with those of previous studies. The cause of the increased kidney volume due to AKI may be edema or inflammation of the renal parenchyma [7,9,60]. Importantly, kidney enlargement occurred despite overall body weight loss, highlighting its role as a disease-specific indicator of fluid accumulation. This indicator, kidney volume, also reflects renal disease transitions and may allow for the evaluation of the recovery or worsening of renal impairment among the same individuals [7,9,35]. The significant increase in kidney volume due to renal injury observed in this study suggests that kidney volume may be an indicator of renal status for drug-induced AKI.

This study had some limitations. First, consensus on the optimal combination of parameters for AKI is lacking. Therefore, T_1_, T_1rho_, T_2_, and T_2_* maps were used in this study, whereas diffusion-weighted imaging and arterial spin labelling were used in previous studies to evaluate AKI [4,9,13,33,35,36]. Multiparametric MRI has the disadvantage of longer scan times; however, it also has the advantage of revealing multiple renal characteristics in a single scan by combining them. Hence, the optimal combination of imaging methods remains to be investigated.

Second, only one scan was performed in both groups, which did not allow for early or follow-up observations of AKI. A previous study reported that the relaxation time measurement method can detect renal injury earlier and more accurately than blood tests [17]. Therefore, we would like to increase the number of scanning points per group and investigate when each relaxation time measurement method can detect drug-induced AKI, as well as an effective evaluation method for the early detection of renal injury.

Thirdly, as it was difficult to manually distinguish the OSOM in this study, it was challenging to measure the relaxation times for the cortex and inner stripe of the outer medulla (ISOM). Previous studies have reported that renal injury causes significant changes in the OSOM and cortical layers on T_1_ and T_2_ maps [17,24,25]. Therefore, analyzing images with objectivity and reproducibility and considering methods that can separate the kidney into the cortex, OSOM, and ISOM are necessary.

In contrast to the cortex, changes in medullary T_2_, T_2_*, and T_1rho_ values did not reach statistical significance in our study. This may partly reflect the regional susceptibility of gentamicin toxicity, which primarily targets proximal tubules in the cortex and OSOM [6,12], leaving the medulla less affected during the acute phase. In addition, the medulla is structurally heterogeneous, comprising vascular bundles, loops of Henle, and collecting ducts, which contribute to higher signal variability and partial volume effects at the current spatial resolution. These technical limitations may obscure subtle medullary alterations. Future work using higher resolution imaging or improved segmentation methods may clarify whether medullary changes become more apparent with optimized techniques.

In addition, we did not perform collagen-specific staining, such as trichrome, which would be required to substantiate fibrosis. This represents a limitation of the current acute study and will be addressed in future chronic models.

Although T_1_, T_2_, T_2_*, and T_1rho_ relaxation times are governed by distinct magnetization decay mechanisms (spin–lattice, spin–spin, susceptibility-related dephasing, and spin-lock relaxation, respectively), in our study, they all demonstrated broadly similar increases in the renal cortex following gentamicin-induced AKI. This convergence likely reflects the fact that the predominant pathological processes—edema, altered water distribution, tubular damage, and extracellular matrix remodeling—affect multiple relaxation pathways simultaneously. For example, increased water content and structural disorganization can prolong both T_1_ and T_2_, while collagen deposition and macromolecular interactions influence T_1rho_, and edema-related magnetic susceptibility changes can extend T_2_*. Thus, although the underlying physics differ, the shared pathological substrates of AKI yield parallel changes across these relaxation parameters. This suggests that multiparametric MRI can provide a consistent and reinforcing picture of renal injury while also offering complementary sensitivity to specific tissue properties.

## 5. Conclusions

We used multiparametric MRI to evaluate renal morphology and function in a drug-induced AKI model. The results showed that the T_1_ map is the most sensitive quantitative evaluation of any renal injury, the T_1rho_ map can detect changes in macromolecular products leading to fibrosis, the T_2_ map can sensitively evaluate renal edema, and the T_2_* map can evaluate changes due to oxygenation and water content. This study demonstrated that combining these techniques is a noninvasive and effective way to assess drug-induced AKI.

## Figures and Tables

**Table 1:** Correlations between relaxation time and blood tests, where A is the correlation in the renal cortex and B is the correlation in the renal medulla. The T_1_ map in the cortex is positively correlated with Na, blood urea nitrogen, creatinine, and hematocrit, which significantly increased in the disease model (A).

**(A)**				
	**T_1_ map**	**T_1rho_ map**	**T_2_ map**	T_2_* map
**Na**	0.75	0.50	0.46	0.12
**K**	−0.19	−0.43	−0.41	−0.34
**Cl**	0.27	0.43	0.59	0.49
**BUN**	0.68	0.57	0.59	0.45
**Crea**	0.70	0.45	0.46	0.25
**Hct**	0.83	0.57	0.54	0.17
(**B**)				
	**T_1_ map**	**T_1rho_ map**	**T_2_ map**	T_2_* map
**Na**	0.43	0.27	0.59	−0.05
**K**	−0.31	−0.44	−0.47	−0.33
**Cl**	0.24	0.46	0.59	0.39
**BUN**	0.51	0.43	0.28	0.31
**Crea**	0.41	0.22	−0.01	0.10
**Hct**	0.65	0.47	0.19	0.01

## Data Availability

The original contributions presented in this study are included in the article. Further inquiries can be directed to the corresponding author.
